# Immunogenicity and Cross-Reactivity of 2009–2010 Inactivated Seasonal Influenza Vaccine in US Adults and Elderly

**DOI:** 10.1371/journal.pone.0016650

**Published:** 2011-01-31

**Authors:** Hang Xie, Xianghong Jing, Xing Li, Zhengshi Lin, Ewan Plant, Olga Zoueva, Hong Yang, Zhiping Ye

**Affiliations:** 1 Laboratory of Respiratory Viral Diseases, Division of Viral Products, Office of Vaccine Research and Review, United States Food and Drug Administration, Bethesda, Maryland, United States of America; 2 Office of Biostatistics and Epidemiology, Center for Biologics Evaluation and Research, United States Food and Drug Administration, Bethesda, Maryland, United States of America; Hallym University, Republic of Korea

## Abstract

The campaign of 2009–2010 Northern Hemisphere seasonal vaccination was concurrent with the 2009 H1N1 pandemic. Using a hemagglutination inhibition (HAI) assay, we evaluated the immunogenicity and cross-reactivity of 2009–2010 inactivated trivalent influenza vaccine (TIV) in US adult and elderly populations. Vaccination of TIV resulted in a robust boost on the antibody response of all subjects to seasonal A/Brisbane/59/2007 (H1N1) and A/Uruguay/716/2007 (H3N2) with over 70% of recipients reaching a seroprotective titer of 40. B/Brisbane/60/2008 was the least immunogenic among the three seasonal vaccine strains with <30% of TIV recipients reaching a seroprotective titer of 40. TIV vaccination also induced a moderate boost on the pandemic specific antibody responses. Twenty-four percent of adults and 36% of elderly reached a seroprotective HAI titer of 40 or more against pandemic A/South Carolina/18/2009 (H1N1) after receiving TIV compared to 4% and 7% at the beginning of vaccination, respectively. In addition, 22% of adults and 34% of elderly showed an increase of 4-fold or more in A/South Carolina/18/2009 specific HAI titers after TIV vaccination. The pandemic specific cross-reactive antibodies strongly correlated with the post-vaccination HAI titers against the seasonal H3N2 vaccine strain in all subjects.

## Introduction

The newly emerged 2009 pandemic H1N1 viruses have the hemagglutinin (HA) gene derived from the classical swine lineage, and are genetically and antigenically distinguished from recently circulating seasonal H1N1 influenza viruses [1]. The majority of the population had no prior exposure to the 2009 pandemic H1N1 viruses and thus had little pre-existing immunity against these viruses except those over the age of 60 years [2]. Despite the dominance of 2009 pandemic H1N1 viruses in the Northern Hemisphere, sporadic infections caused by seasonal influenza viruses were also reported. Hence, WHO and CDC recommended that the public seek both seasonal and pandemic vaccines for the 2009–2010 flu season. Thanks to extensive media coverage of the 2009 pandemic and heightened public awareness of the potential risk of influenza, a quite percentage of people have followed the recommendation and taken the 2009–2010 seasonal vaccine before the pandemic monovalent vaccine became widely available. However, the effectiveness of this vaccination strategy was unclear, especially with regard to potential impact on prevention of 2009 H1N1 pandemic. In this study, we evaluated the immunogenicity of 2009–2010 Northern Hemisphere inactivated trivalent influenza vaccine (TIV) and its effects on the development of cross-reactive antibody response to the current pandemic influenza as measured by hemagglutination inhibition (HAI) assay.

## Results

### Immunogenicity of 2009–2010 seasonal TIV

**Figure 1:**
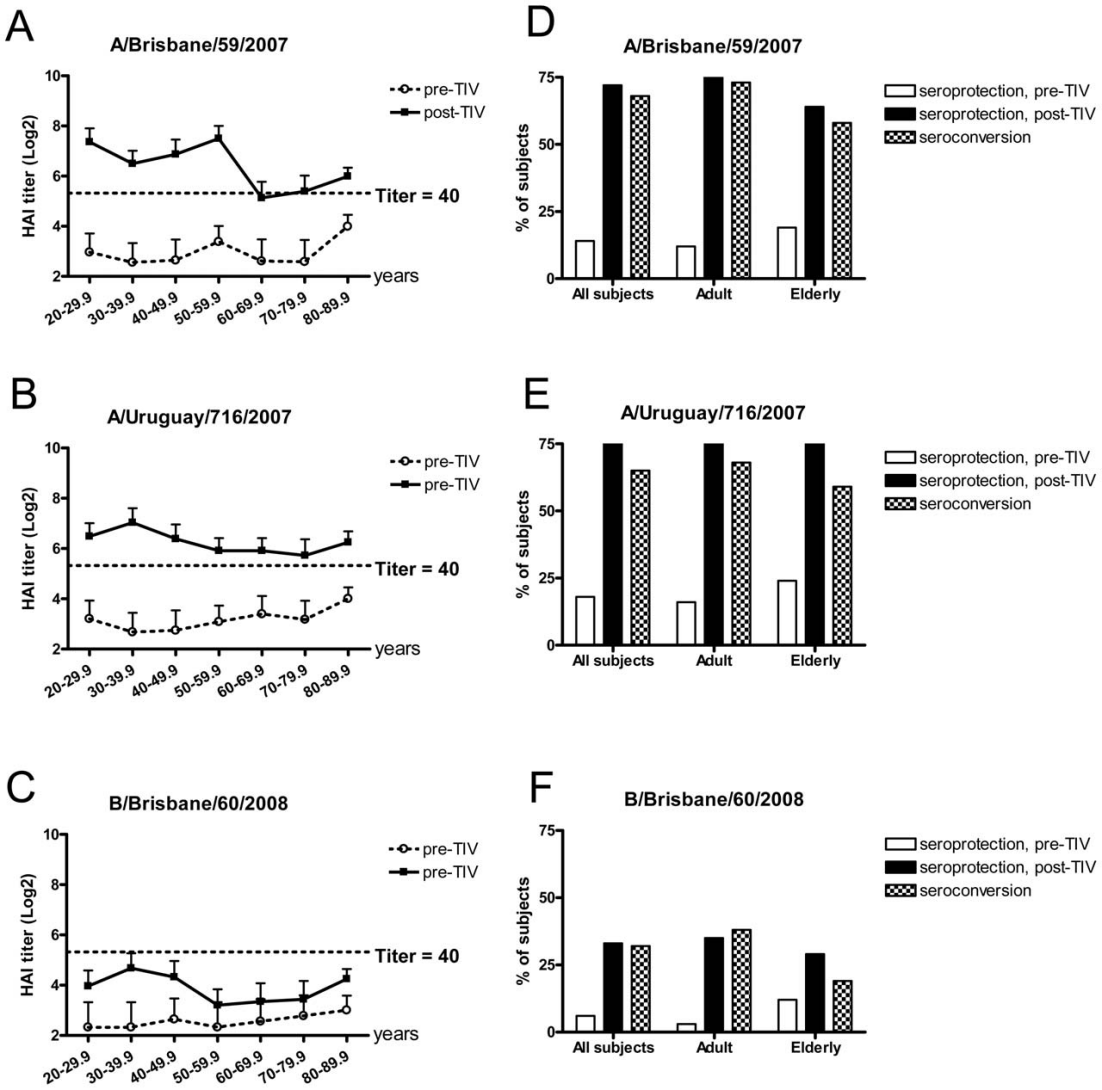
Hemagglutination inhibition (HAI) titers against 2009–2010 seasonal vaccine strains. Serum samples collected from US healthy volunteers vaccinated with 2009–2010 inactivated unadjuvanted trivalent influenza vaccine (TIV) were tested by HAI assay using 0.5% turkey erythrocytes. The geometric mean titers (GMTs), the seroprotection rates (the proportion of subjects having an HAI titer ≥40) before and 21 days after TIV administration, and the seroconversion rates (the proportion of subjects having a ≥4-fold increase in HAI titers) were plotted according to the age distribution of vaccinees for A/Brisbane/59/2007 (H1N1) (A and D), A/Uruguay/716/2007 (H3N2) (B and E), and B/Brisbane/60/2008 (C and F), respectively. The 95% CI for individual HAI GMTs are shown as error bars. The dotted lines indicate HAI titer of 40.

There were no significant differences in the baseline geometric mean of HAI titers (GMTs ≤20) among all the subage groups against each of the three current seasonal vaccine strains (A/Brisbane/59/2007 (H1N1), A/Uruguay/716/2007 (H3N2), and B/Brisbane/60/2008) at the beginning of enrollment (Figure 1A, 1B and 1C). Administration of 2009–2010 seasonal TIV induced a robust antibody response in adults (20.1–64.8 years old) and elderly (65.4–88.2 years old) toward the type A vaccine strains (Figure 1A and 1B). Overall, >70% of all subjects had an HAI titer of 40 against both H1 and H3 seasonal strains after receiving TIV and ≥65% of them showed a 4-fold or more increase in the post-vaccination titers (Figure 1D and 1E). An HAI titer of 40 has been suggested as a seroprotective measure associated with at least 50% reduction in the risk of influenza infection or diseases in humans [3]–[5]. On the contrary, both adult and elderly groups responded significantly less robustly to B/Brisbane/60/2008 than to the other two type A vaccine strains after TIV vaccination (Figure 1C and 1F). Fewer than 35% of the TIV recipients reached a seroprotective titer of 40 or more toward B/Brisbane/60/2008. Only 38% of adults and 19% of elderly showed a ≥4-fold rise in B/Brisbane/60/2008 specific HAI titers after TIV vaccination (Figure 1F).

### Cross-reactive HAI responses before and after 2009–2010 seasonal TIV vaccination

**Figure 2:**
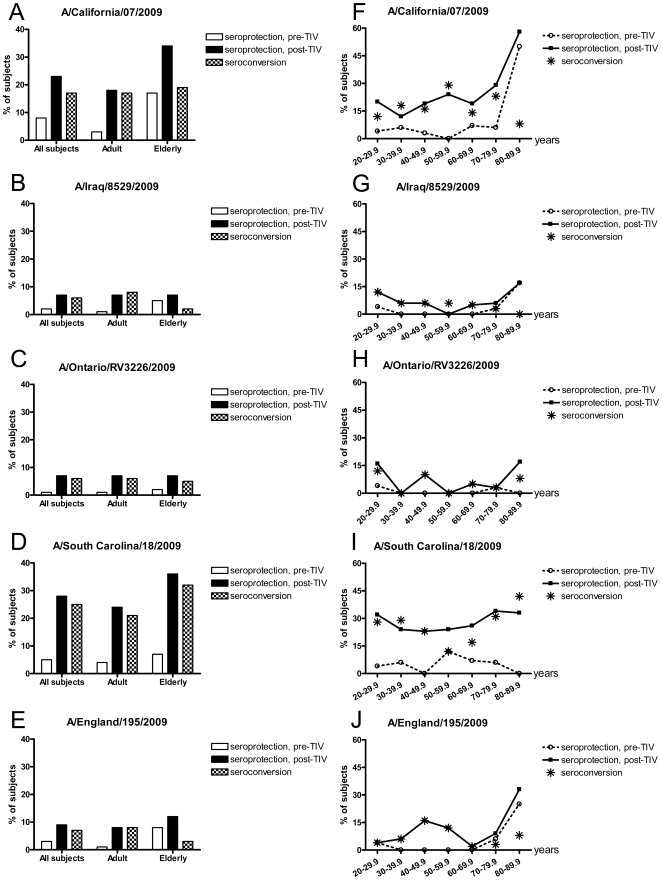
Hemagglutination inhibition (HAI) titers against 2009 pandemic H1N1 viruses. Serum samples collected from US healthy volunteers vaccinated with 2009–2010 inactivated unadjuvanted trivalent influenza vaccine (TIV) were tested for cross-reactivity against 2009 pandemic H1N1 viruses by HAI assay using 0.5% turkey erythrocytes. The pandemic influenza specific seroprotection rates (the proportion of subjects having an HAI titer ≥40) before and 21 days after TIV administration and the seroconversion rates (the proportion of subjects having a ≥4-fold rise in HAI titers) were plotted according to the age distribution of vaccinees for A/California/07/2009 (H1N1) (A and F), A/Iraq/8529/2009 (H1N1) (B and G), A/Ontario/RV3226/2009 (H1N1) (C and H), A/South Carolina/18/2009 (H1N1) (D and I) and A/England/195/2009 (H1N1) (E and J), respectively. * indicates the corresponding seroconversion rate in panel F, G, H, I, and J.

The campaign of 2009–2010 seasonal TIV vaccination coincided with the prevalence of 2009 H1N1 pandemic. Hence, we were interested in how the current seasonal TIV vaccination impacted the cross-reactive antibodies against 2009 pandemic H1N1 viruses including A/California/07/2009 (the vaccine strain for 2009 pandemic monovalent vaccine) and its four recent variants (A/Iraq/8529/2009, A/England/195/2009, A/Ontario/RV3226/2009 and A/South Carolina/18/2009). At the beginning of enrollment, fewer than 10% of the participants of all ages had detectable pre-existing antibodies against 2009 pandemic H1N1 viruses (Figure 2A, 2B, 2C, 2D, and 2E).But 17–50% of elderly at 80 years or older showed a baseline HAI titer ≥40 against pandemic A/California/07/2009, A/Iraq/8529/2009 and A/England/195/2009, but not toward A/Ontario/RV3226/2009 and A/South Carolina/18/2009 (Figure 2F, 2G, 2H, 2I and 2J).

**Figure 3:**
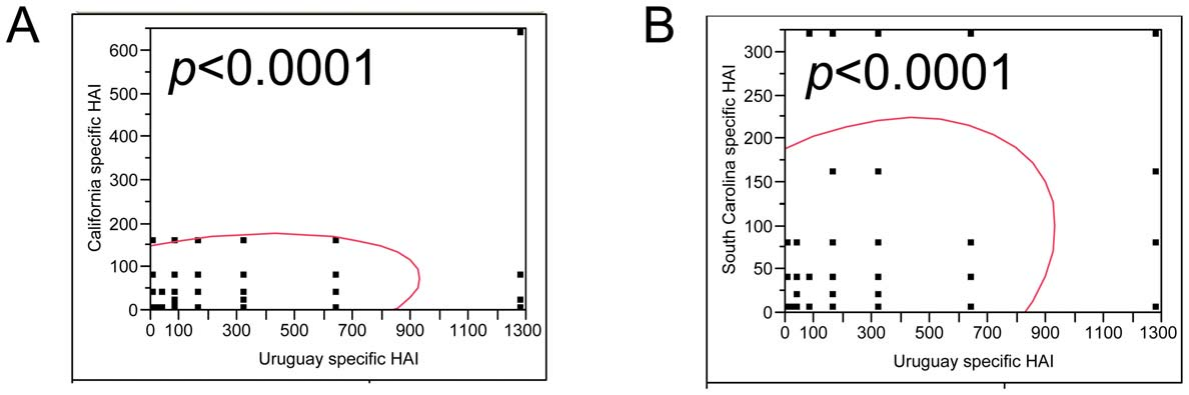
Correlation of antibody titers specific for seasonal vaccine strains and pandemic viruses after seasonal vaccination. Serum samples were collected on 21 days after administration of 2009–2010 inactivated unadjuvanted trivalent influenza vaccine (TIV) in US healthy adults and elderly. An HAI assay was performed using 0.5% turkey erythrocytes. A correlation analysis between seasonal strain specific HAI titers and pandemic strain specific HAI titers was performed using nonparametric Spearman's ρ test by JMP Version 7. The scatterplots of HAI titers are shown in the presence of 95% bivariate normal density ellipses (indicating the distribution of 95% of individual data points plotted) and corresponding *p* values. A, A/California/07/2009 (H1N1) vs A/Uruguay/716/2007 (H3N2); B, A/South Carolina/18/2009 (H1N1) vs A/Uruguay/716/2007 (H3N2).

Surprisingly, both adult and elderly groups simultaneously showed an increased antibody response to pandemic A/California/07/2009 and A/South Carolina/18/2009 after receiving 2009–2010 seasonal TIV, and at a less extent to the other three pandemic variants including A/Iraq/8529/2009, A/Ontario/RV3226/2009 and A/England/195/2009 (Figure 2A, 2B, 2C, 2D, and 2E). The most notable rise occurred in the post-vaccination antibody response to A/South Carolina/18/2009 (H1N1), a recently isolated pandemic H1N1 variant (Figure 2D and 2I). Twenty-four percent of adults and 36% of elderly reached a seroprotective titer of 40 against A/South Carolina/18/2009 (H1N1) after TIV vaccination compared to 4% and 7% at the enrollment respectively (Figure 2D). Additionally, 22% of adults and 34% of elderly showed a≥4-fold increase in A/South Carolina/18/2009 specific HAI titers after receiving TIV (Figure 2I). In particular, 42% of the subjects born before 1930 showed increased antibody response to A/South Carolina/18/2009 by a factor of 4 or more following TIV vaccination (Figure 2I). The cross-reactive antibody responses to A/California/07/2009 (H1N1) and A/South Carolina/18/2009 (H1N1) after TIV vaccination were comparable to that induced by B/Brisbane/60/2008 (Figure 1F, 2A and 2D). A correlation analysis indicated that post-vaccination HAI titers against pandemic A/California/07/2009 and A/South Carolina/18/2009 strongly correlated with those against seasonal A/Uruguay/716/2007 (H3N2) in all TIV recipients (*p*<0.0001, Figure 3A and 3B). The same correlations were observed between the post-vaccination HAI titers against the other three pandemic viruses and seasonal A/Uruguay/716/2007 (H3N2) (data not shown).

## Discussion

The effectiveness of seasonal vaccines largely relies on how well selected vaccine strains represent circulating influenza viruses in the environment. Antigenically poorly matched vaccine strains could lead to significantly reduced protection in vaccinated population as shown in the 2007–2008 flu season [6]. Hence, it is necessary to continue annual evaluation of influenza vaccine strains as currently led by WHO through its collaborative centers over the world. As one of the WHO collaborative centers, each year our laboratory will prescreen human vaccinated serum samples collected from US seasonal clinical trials and select those having post-vaccination titers ≥40 against all three vaccine strains for distribution to other collaborative centers for further evaluation. In January of 2010, 24 pairs (before and after seasonal vaccination) each of selected human clinical samples representing adult and elderly populations in US were distributed for annual vaccine strain evaluation. Because of the predominance of 2009 pandemic H1N1 viruses in circulations, however, the antibody responses to the seasonal H1 vaccine strain were not considered [7]. By the same token, the cross-reactivity of the 2009–2010 seasonal TIV toward the pandemic H1N1 viruses was not evaluated [7].

But it does not necessarily imply that the 2009–2010 seasonal TIV played no role in protecting against 2009 H1N1 pandemic, since a suboptimal vaccine could still provide some protection against influenza-related hospitalization, especially in persons with high risk medical conditions [8], [9]. In addition, a good percentage of US population has received 2009–2010 seasonal TIV before the pandemic monovalent vaccine became widely available. Thus, it was of value to evaluate the impact of seasonal vaccination on the development of cross-reactive antibodies against pandemic viruses. With the expanded sample sizes including all the subjects enrolled in US seasonal trials, we showed in the present study that both adult and elderly groups had increased antibody responses to pandemic vaccine strain A/California/07/2009 (H1N1) and a newly isolated variant A/South Carolina/18/2009 (H1N1) after receipt of 2009–2010 TIV. This is unlikely due to prior infection of 2009 pandemic since the majority of the subjects born after 1930 had a very low baseline titer (<10) against these viruses. On the other side, concurrent pandemic infection during the seasonal TIV vaccination might not be the cause either, otherwise the subjects at 80 years or older would not respond to A/South Carolina/18/2009, since 50% of them already had seroprotective antibody titers against the pandemic vaccine strain A/California/07/2009 before TIV vaccination. Hence, the moderate boost on pandemic specific antibody responses observed in the current study was more likely resulted from 2009–2010 TIV vaccination. Lee et al. (2010) also reported that the 2009–2010 Southern Hemisphere seasonal TIV induced a certain degree of cross-reactive antibodies in healthy young military recruits against a local 2009 H1N1 pandemic isolate [10]. This suggested 2009–2010 TIV vaccination regardless of Northern or Southern Hemisphere formulation could provide some cross-immunity against 2009 H1N1 pandemic at least in adults. This was new because Hancock et al. (2009) reported that seasonal vaccinations prior to 2009 induced little or no cross-reactive antibodies against pandemic H1N1 viruses [11]. However, Plennevaux et al. (2009) also reported that the adults with seasonal influenza vaccination during the 2004–2009 periods had significantly higher HAI titers and seroprotection rates against 2009 pandemic than the subjects without prior seasonal vaccinations [12]. Using a highly sensitive pseudovirus based influenza HA neutralization assay, Labrosse et al. (2010) found that the subjects with 2008–2009 seasonal vaccination showed significantly higher neutralizing titers against pandemic A/California/04/2009 (H1N1) than the subjects without the same seasonal vaccination [13]. They also found a strong correlation between the neutralizing titers against A/Brisbane/59/2009 (H1N1) and A/California/04/2009 (H1N1) [13]. Interestingly, we found in the present study that the post-vaccination HAI titers against A/California/07/2009 (H1N1) and A/South Carolina/18/2009 (H1N1) strongly correlated with their seasonal H3 specific antibody response instead seasonal H1 specific antibodies following 2009–2010 TIV administration. Additionally, it was out of many experts' initial anticipation that a single 15 µg HA dose of pandemic monovalent vaccine was found to be sufficient enough to induce a robust antibody response and a priming-boosting regimen was unnecessary in most of vaccinees, despite these swine-origin H1N1 viruses were new to the public [12], [14], [15]. This may be partially attributed to the priming effect of the current and prior seasonal vaccinations, since a substantial portion of subjects enrolled in the 2009 pandemic monovalent clinical trials had already received the 2009–2010 seasonal vaccine [14].

A moderate boost on cross-reactive response to a novel influenza virus by seasonal vaccination may not lead to a complete protection against a pandemic, but might reduce the burden of infections substantially in affected subjects, which is often seen in young children receiving partial vaccination [16]–[18]. A surveillance study on military service members stationed in the US soil has found that prior seasonal vaccinations between 2004–2009 were positively associated with protection against clinically apparent and laboratory-confirmed 2009 pandemic H1N1 illness [19]. This study along with another study led by L. Garcia-Garcia in Mexico City also found that the cross-protective effect of seasonal vaccination was more obvious in subjects with severe symptoms or hospitalization than those with mild outcome [20]. Controversies also exist. The Canadian public petitioner D.M. Skowronski and her team have claimed that an increased risk of 2009 pandemic H1N1 illness was present in local communities after receipt of seasonal vaccines [21], [22]. However, the bias or confounding factors could not be ruled out as these always being the major concern for observational studies. Interestingly, another cohort study on hospital nurses from Ontario, Canada has reported a possible positive effect of the 2008–2009 TIV on reducing risk of 2009 pandemic infections though the sample size was small [23]. A more recent study by Cowling et al. (2010) has found no direct link between the 2008–2009 Southern Hemisphere seasonal TIV and increased risk of 2009 H1N1 pandemic infection in children at ages of 6–15 years from Hong Kong [24].

In addition to the pandemic specific cross-reactive antibody responses elicited by 2009–2010 TIV, our data also indicated that B/Brisbane/60/2008 was significantly less immunogenic than the other two type A vaccine components that, only 33% of adult and elderly subjects reached a seroprotective titer of 40 or more against B/Brisbane/60/2008 after TIV vaccination. The low immunogenicity of B/Brisbane/60/2008 was likely due to that it was a new strain unlike A/Brisbane/59/2007 (H1N1) and A/Uruguay/716/2007 (H3N2) (an A/Brisbane/10/2007-like virus) that have been vaccine components since 2008–2009 season. However, repeated immunization may overcome this problem since B/Brisbane/60/2008 remains as a vaccine component for the 2010–2011 influenza season in the Northern Hemisphere.

Nevertheless, ours and others' studies suggest that annual seasonal vaccination play an important role in protecting the public not only against seasonal flu but also a pandemic. In the United States, the seasonal influenza vaccination coverage jumped significantly during the 2009–2010 flu season, especially in children and adults aged 18–49 years without high risk medical condition [25], which should be greatly attributed to the joint campaign by governments and media. Continuous seasonal vaccination on individuals and expanding vaccination coverage in the community could potentially reduce disease burden from routinely circulating or newly emerging influenza viruses in future.

## Materials and Methods

### Ethics statement

All the human pre- and post-vaccinated serum samples involved in the present study were analyzed anonymously at CBER/FDA.

### Vaccinated human serum samples

Paired serum panels (pre-seasonal vaccination and post-seasonal vaccination) from 2009–2010 TIV vaccinated healthy human volunteers were kindly provided by Dr. Mark Blatter (Primary Physicians Research, Inc., Jefferson Hills, PA), Dr. Michael J. Noss (Radiant research, Cincinnati, OH), and Dr. Rex Biedenbender (Eastern Virginia Medical School, Norfolk, VA) respectively in support of WHO consultation on the composition of influenza vaccine for the Northern Hemisphere. A written informed consent was provided to all volunteers at the beginning of the enrollment by each physician according to the guidelines of the corresponding institutional review board. Unless otherwise indicated, 120 adults (20.1–64.8 years old) and 59 elderly (65.4–88.2 years old) from US were given a single dose (15 µg HA/strain/dose) of 2009–2010 unadjuvanted TIV intramuscularly (Table 1). The TIV product mentioned above was formulated according to Northern Hemisphere seasonal vaccine composition containing A/Brisbane/59/2007 (H1N1), A/Uruguay/716/2007 (H3N2), and B/Brisbane/60/2008) as recommended by WHO. Sera were collected at the time of enrollment and 21 days after TIV vaccination.

**Table 1:** Demographic characteristics of the subjects vaccinated with 2009–2010 seasonal inactivated trivalent vaccine.

Characteristic	20–29.9 Year	30–39.9 Year	40–49.9 Year	50–59.9 Year	60–69.9 Year	70–79.9 Year	80–89.9 Year
No. of subjects	25	17	31	17	42	35	12
Median age	24.6 years	36.7 years	45.2 years	55.2 years	63.5 years	73.4 years	83.4 years

NOTE. The 2009–2010 Northern Hemisphere inactivated trivalent influenza vaccine (TIV) contained 15 µg of hemagglutinin of each of the following strains: A/Brisbane/59/2007 (H1N1), A/Uruguay/716/2007 (H3N2), and B/Brisbane/60/2008.

### Hemagglutination inhibition (HAI) assay

The 2009–2010 seasonal TIV vaccine strains A/Brisbane/59/2007 (H1N1), A/Uruguay/716/2007 (H3N2), and B/Brisbane/60/2008 and the 2009 H1N1 pandemic viruses A/California/07/2009, A/Iraq/8529/2009, A/Ontario/RV3226/2009, A/South Carolina/18/2009, and A/England/195/2009 were propagated in 9-day-old specific pathogen-free embryonated chicken eggs. The immunogenicity of 2009–2010 seasonal TIV was evaluated by HAI assay using 4 HA units of viruses and 0.5% turkey erythrocytes according to the procedures described previously [26], [27]. Sera were pre-treated with receptor-destroying enzyme (RDE, Denka-Seiken, Tokyo, Japan) at 37°C for 18 h followed by heat-inactivation at 56°C for another 30 min. RDE-treated sera were then serially two-fold diluted starting from 1∶10 dilution prior to HAI assay. A titer of 5 was assigned if negative HAI reactions occurred at ≥1∶10.

### Data analysis and statistics

All subjects including adults and elderly were mixed together and grouped according to the ages with 10-year-intervals with demographic characteristics shown in Table 1. Three immunogenicity end points were chosen for the final data analysis: the geometric mean of HAI titers (GMTs); the seroprotection rate (the proportion of subjects with HAI titers ≥40); and the seroconversion rate (the proportion of subjects with a ≥4-fold rise in HAI titers). A correlation analysis between seasonal strain specific HAI titers and pandemic specific HAI titers after 2009–2010 TIV administration was performed using nonparametric Spearman's ρ test by JMP (Version 7). A *p* value of <0.05 was considered statistically significant.
